# Definitions

**Published:** 1877-02-20

**Authors:** 


*Definitions.*—The word *prescription* is from the Latin *præ*, before; *scribo*, I write; and is applied to the written directions of a physician for the preparation and use of remedies. A prescription may or may not refer to drugs—the physician may prescribe bathing, exercise, blood-letting, etc. It is, therefore, more comprehensive than the word *formula*, from the Latin *forma*, a form, which is only applied to the form or directions given for the preparation and use of pharmaceutical remedies or drugs.

The symbol *R.* is an abbreviation of the Latin *recipe*, take thou, and is usually placed at the beginning of every formula The Latin *M* attached to formulæ is an abbreviation of *misce*, mix thou, and the letter *S* an abbreviation for *signatura, the signature*, or the directions to be written upon the label as a guide to the patient in the use of the medicine.

